# Supplementary material for: ROS regulation of stigma papillae growth and maturation in Arabidopsis thaliana
Source: Plant Reprod. 2025 Jun 5;38(2):14. doi: 10.1007/s00497-025-00524-2 (PMC12137503; doi:10.1007/s00497-025-00524-2)
Supplement: Supplementary file 1 — Supplementary file1 (PDF 13,420 KB) [file 497_2025_524_MOESM1_ESM.pdf]

Figure S1

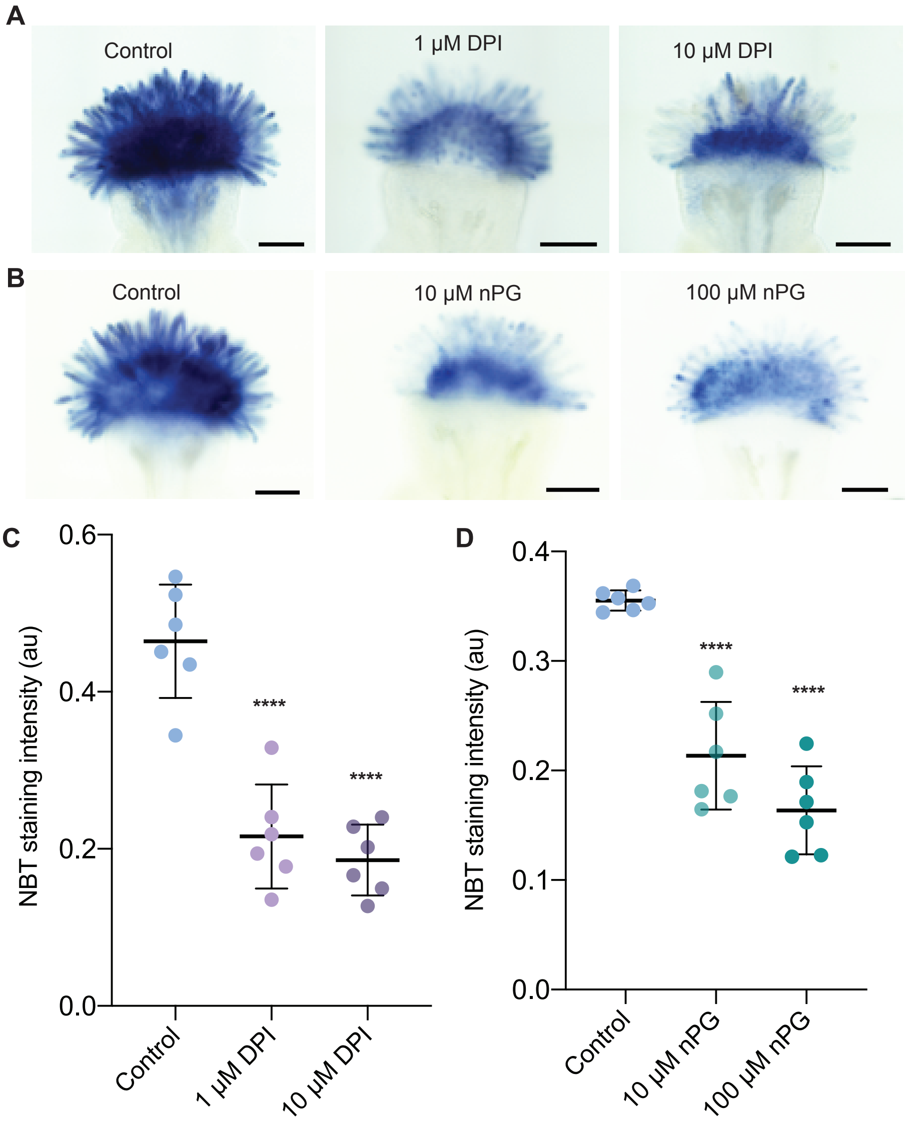
A
Control
1 μM DPI
10 μM DPI
B
Control
10 μM nPG
100 μM nPG
C
0.6
0.4
0.2
0.0
NBT staining intensity (au)
****
****
Control
1 μM DPI
10 μM DPI
D
0.4
0.3
0.2
0.1
0.0
NBT staining intensity (au)
****
****
Control
10 μM nPG
100 μM nPG

Figure S2

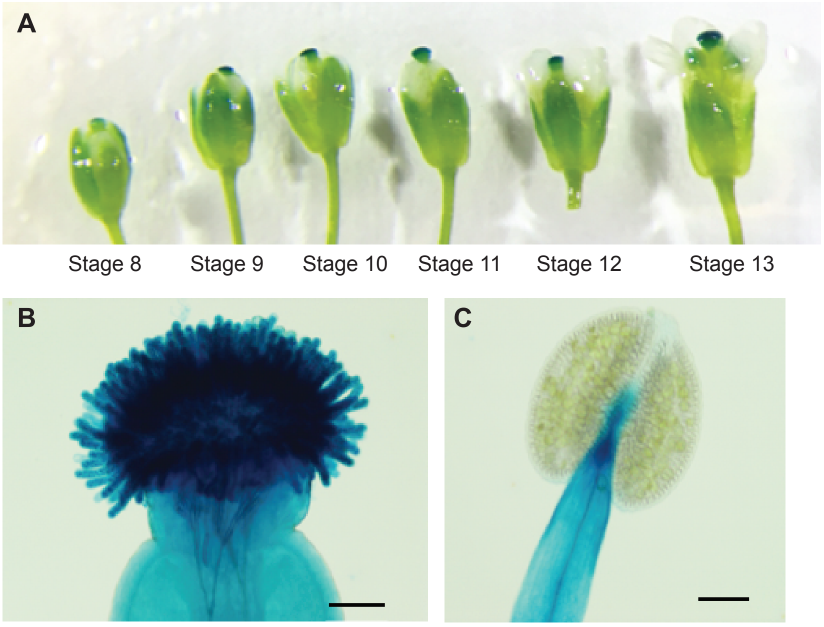

Figure S3

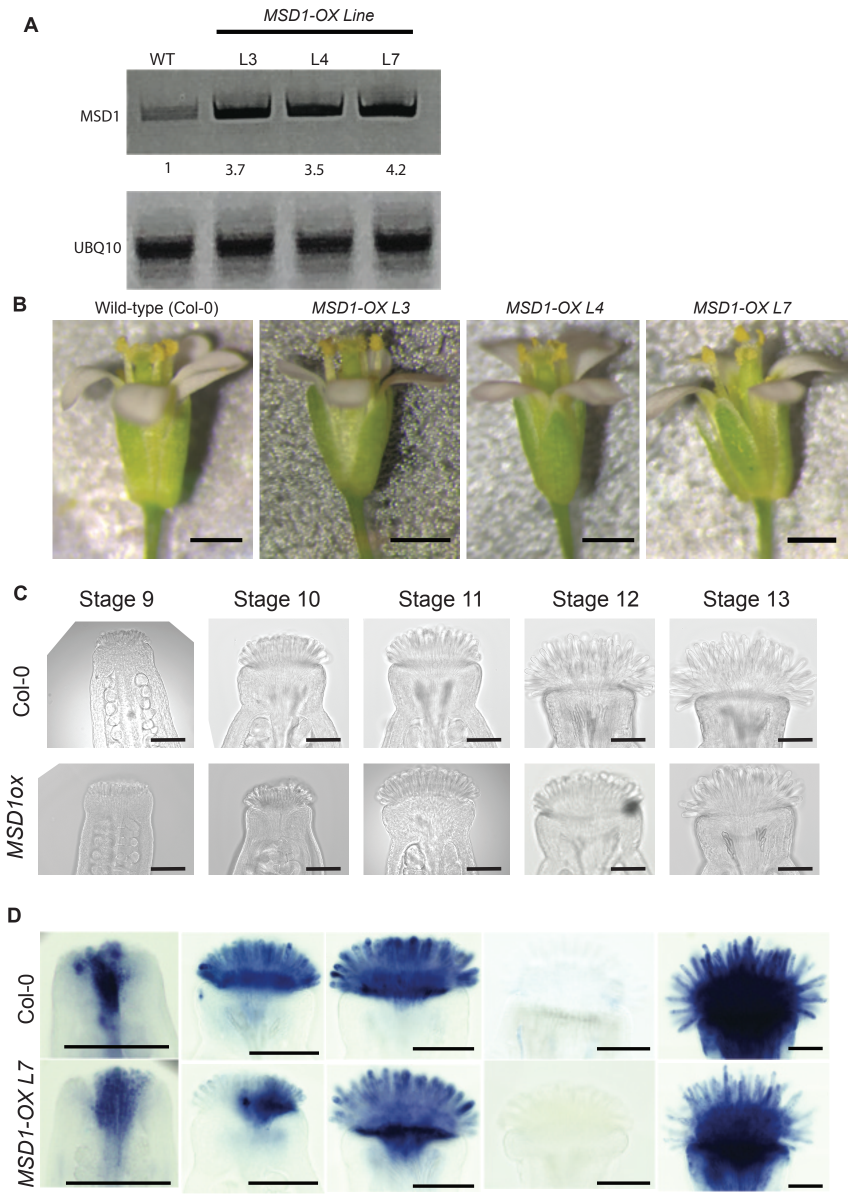
A
MSD1-OX Line
WT
L3
L4
L7
MSD1
1
3.7
3.5
4.2
UBQ10
B
Wild-type (Col-0)
MSD1-OX L3
MSD1-OX L4
MSD1-OX L7
C
Stage 9
Stage 10
Stage 11
Stage 12
Stage 13
Col-0
MSD1ox
D
Col-0
MSD1-OX L7

Figure S4

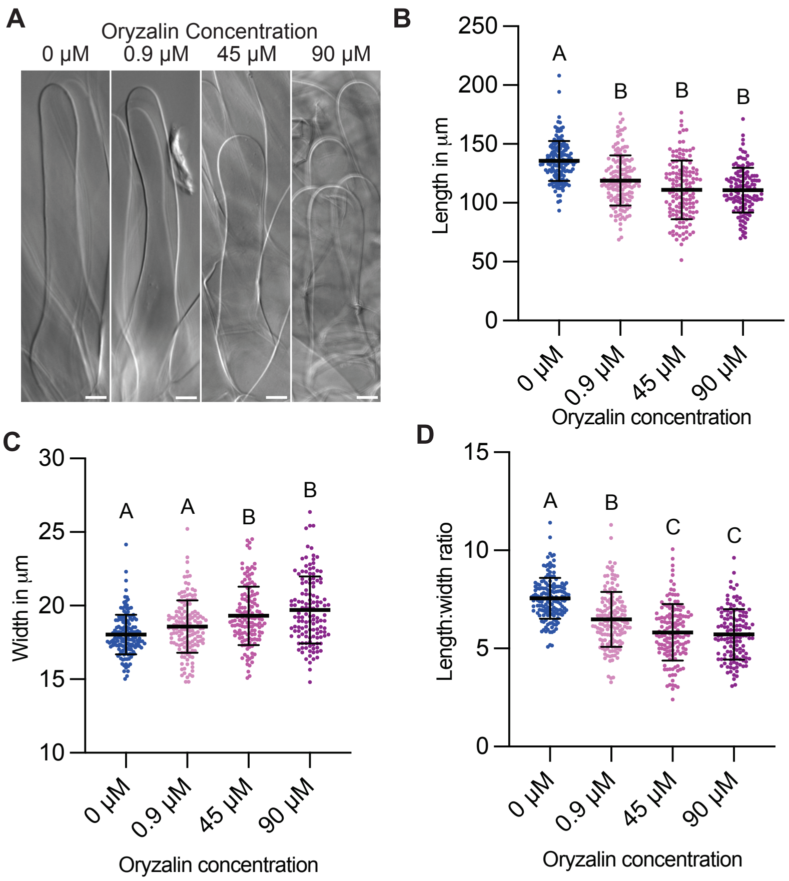
A
Oryzalin Concentration
0 μM
0.9 μM
45 μM
90 μM
B
Length in μm
250
200
150
100
50
0
A
B
B
B
0 μM
0.9 μM
45 μM
90 μM
Oryzalin concentration
C
Width in μm
30
25
20
15
10
A
A
B
B
0 μM
0.9 μM
45 μM
90 μM
Oryzalin concentration
D
Length:width ratio
15
10
5
0
A
B
C
C
0 μM
0.9 μM
45 μM
90 μM
Oryzalin concentration

Supplemental Table 1. Primers used for cloning and RT-PCR

Primer Name	Sequence
**pAT5G53710_F**	5’GGGGACAAGTTTGTACAAAAAAGCAGGCTTCACCGGTGCTTGTATCTTTTTAGAGCTC3’
**pAT5G53710_R**	5’GGGGACCACTTTGTACAAGAAAGCTGGGTGCATTACTGTTTTCGTTATTGGAAGC3’
**InF-AT5G53710-pGWB554_F**	5’GACCAAAGGGCAATTGGGTGCTTGTATCTTTTTAGAGCTC3’
**InF-AT5G53710-pGWB554_R**	5’TGTTGATAACTCTAGACATTACTGTTTTCGTTATTGGAAGC3’
**RT-At3g10920-F**	5’ATGGCGATTCGTTGTGTAGCGAG3’
**RT-At3g10920-R**	5’GGAACTGGTTCATCTCCTTATGTC3’
**RT-Ubq10-F**	5’TTTGTGTTTTGGGGCCTTGT3’
**RT-Ubq10-F**	5’AACAGCTCAACACTTTCGCT3’
